# Stem Cell-Like Properties of CK2β-down Regulated Mammary Cells

**DOI:** 10.3390/cancers9090114

**Published:** 2017-08-31

**Authors:** Eve Duchemin-Pelletier, Megghane Baulard, Elodie Spreux, Magali Prioux, Mithila Burute, Baharia Mograbi, Laurent Guyon, Manuel Théry, Claude Cochet, Odile Filhol

**Affiliations:** 1 Chemistry and Biology Department, Université Grenoble Alpes, F-38400 Grenoble, France; eve_dp87@yahoo.fr (E.D.-P.); megghanebaulard@yahoo.fr (M.B.); elodie.spreux@hotmail.fr (E.S.); magali.prioux@free.fr (M.P.); laurent.guyon@cea.fr (L.G.); claude.cochet@cea.fr (C.C.); 2 Biology of Cancer and Infection, UMRS1036, Inserm, F-38054 Grenoble, France; 3 Biology of Cancer and Infection, Biosciences & Biotechnology Institute of Grenoble, CEA, F-38054 Grenoble, France; 4 Laboratoire de Physiologie Cellulaire et Végétale, Biosciences & Biotechnology Institute of Grenoble, UMR5168, CEA/INRA/CNRS, F-38054 Grenoble, France; mithila.pune@gmail.com (M.B.); manuel.thery@cea.fr (M.T.); 5 Biology Department, Inserm, CNRS, IRCAN, Université Côte d’Azur, F-06000 Nice, France; Baharia.MOGRABI@unice.fr

**Keywords:** protein kinase CK2, stem cell, breast cancer, EMT, epithelial plasticity

## Abstract

The ubiquitous protein kinase CK2 has been demonstrated to be overexpressed in a number of human tumours. This enzyme is composed of two catalytic α or α’ subunits and a dimer of β regulatory subunits whose expression levels are probably implicated in CK2 regulation. Several recent papers reported that unbalanced expression of CK2 subunits is sufficient to drive epithelial to mesenchymal transition, a process involved in cancer invasion and metastasis. Herein, through transcriptomic and miRNA analysis together with comparison of cellular properties between wild type and CK2β-knock-down MCF10A cells, we show that down-regulation of CK2β subunit in mammary epithelial cells induces the acquisition of stem cell-like properties associated with perturbed polarity, CD44^high^/CD24^low^ antigenic phenotype and the ability to grow under anchorage-independent conditions. These data demonstrate that a CK2β level establishes a critical cell fate threshold in the control of epithelial cell plasticity. Thus, this regulatory subunit functions as a nodal protein to maintain an epithelial phenotype and its depletion drives breast cell stemness.

## 1. Introduction

Tumour formation is a complex process that originates from epithelial cells. A wealth of evidence supports the dogma that different events, described by Hanahan and Weinberg as “hallmarks of cancer” participate in tumour progression, from initiation to metastasis [1,2]. Metastasis remains the major cause of cancer-associated death, as cancer cells evade the primary tumour upon tumour microenvironment changes. Thus, a better understanding in the intra- and extra-cellular parameters involved in these processes would be helpful, to identify new targets to fight this scourge. A first mechanism known to allow tumour cells migration is the epithelial-to-mesenchymal transition (EMT), which leads to enhanced cell motility. Several protein kinases are involved in the different molecular signaling circuits that drive EMT. Among them, protein kinase CK2, whose expression is abnormally high in a wide range of tumours, operates as a cancer driver by creating the cellular environment favorable to neoplasia [3]. As a signaling protein, CK2 is a multi-subunit holoenzyme which has many cellular functions associated with a wide repertoire of substrates located in several of cellular compartments. The CK2α catalytic subunits possess a constitutive activity, while the homodimer of CK2β regulatory subunits operate as a regulatory component, modifying the accessibility of binding substrates to the catalytic site of the holoenzyme [4]. Using live-cell fluorescence imaging studies, we previously provided evidence of independent and rapid movement of CK2α and CK2β [5], showing that this kinase can rapidly target specific proteins in response to different stimuli [6].

In mammary epithelial cells, including MCF10A cells, TGFβ induces an EMT by driving expression of specific transcription factors, such as Snail1, Slug, and Twist. Accumulating evidences also suggest that EMT-induced cells exhibited hallmarks commonly attributed to cancer stem cells [7,8]. Recently, we provided evidence that CK2 plays a key role in EMT signaling pathways. More precisely, we showed that in the absence of its CK2β regulatory subunits, the CK2-mediated Snail1 phosphorylation is abrogated, a post-transcriptional modification that is required for Snail1 proteasomal-degradation in epithelial cells. Of note, the expression of CK2α at both transcriptional and protein levels was not affected upon CK2β depletion. Moreover, this CK2β-dependent phosphorylation had a cumulative positive effect on GSK3β-mediated Snail1 phosphorylation. As the phosphorylation of Snail1 participates in its degradation, these results suggest that both kinases can negatively regulate Snail1 stability through its hierarchal phosphorylation. In accordance, we found that in the absence of CK2β, Snail1 was no longer degraded [9]. Another EMT-transcription factor, FoxC2 is retained in the cytoplasm in its CK2-phosphorylated state. However, in the absence of CK2β, FoxC2 cannot be phosphorylated, and enters into the nucleus and activates the transcription of EMT-related genes [10]. Thus, unbalanced expression of CK2 subunits is sufficient to drive epithelial-to-mesenchymal transition, a process involved in cancer invasion and metastasis. This dysregulated expression of CK2 subunits has been observed in breast cancer, renal cancer, lung adenocarcinoma, and glioblastoma [9,11,12,13].

Normal and malignant stem cells share the ability to self-renew while generating differentiated cells [14]. Here, we show that CK2β-deficient MCF10A cells exhibited several properties commonly attributable to cancer stem cells [7,8]. However, we found that though they lacked tumour-initiating ability, they displayed enhanced plasticity and stemness characteristics of normal stem cells. 

## 2. Results and Discussion

### 2.1. ∆CK2β-MCF10A Cells Have Reduced Expression of EMT miRNAs

**Figure 1:**
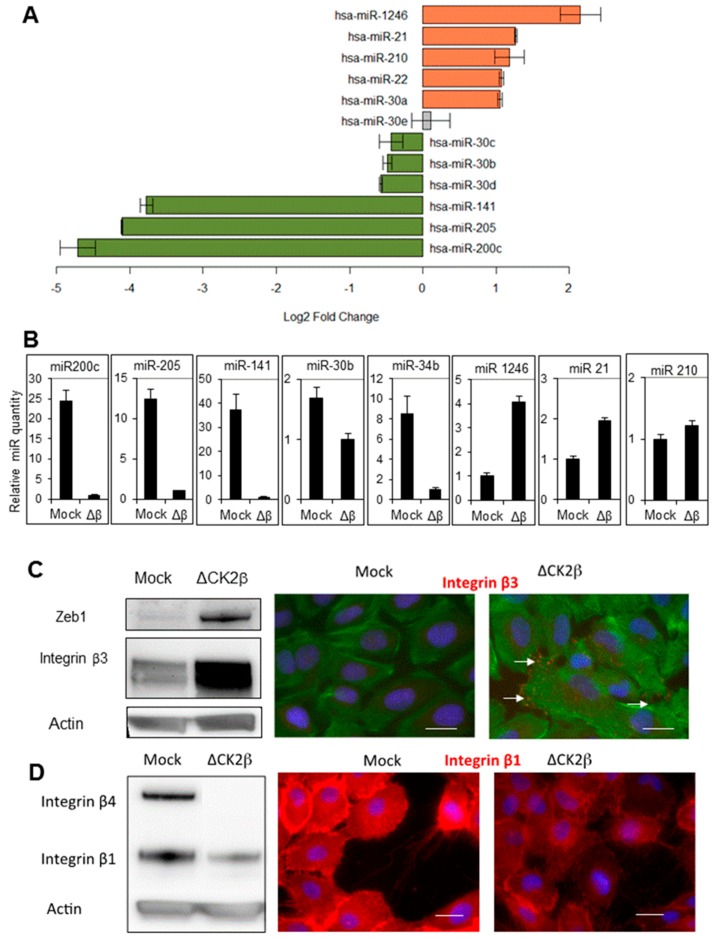
**Modulation of miRNAs in ΔCK2β-MCF10A cells.** (**A**) Log2 fold change of the main miRNAs modulated in CK2β-depleted versus parental MCF10A cells measured by miRNA array analysis; (**B**) Changes of miRNA expression between CK2β-depleted and Mock-MCF10A cells were confirmed by using the indicated TaqMan probes. The relative amount of each miRNAs was determined by cross-normalization to ΔCK2β samples using the comparative method and miR-720 as an internal reference; (**C**) Two targets of miR-200 and miR-30 families, respectively Zeb1 and integrin β3, were analyzed by Western blot and/or immunofluorescence in Mock- and CK2β-depleted cells. The ratio ΔCK2β/Mock of signal intensity in western blot was determined (3.5 and 2.3 for Zeb1 and integrin β3 respectively). Arrows indicate integrin β3 localization; (**D**) Integrin β1 and β4, targets of miR-21 were analyzed by western blot and/or immunofluorescence in Mock- and CK2β-depleted cells. The ratio ΔCK2β/Mock of signal intensity in western blot was 0.4 for integrin β1. F-actin in green, nuclei in blue, and integrin β in red. Scale bar, 10 μm.

Since miRNAs regulate differentiation, cell renewal and invasion [15], we looked for potential changes in miRNA expression that might occur upon CK2β depletion. Using microarrays with the miRNA probe set (Agilent miRNA human V.3), miRNA expression profiles were compared in ∆CK2β- and Mock-MCF10A cells (Table S1). These two cell lines were generated by shRNA-transduction as described in [9], in which less than 10% of the CK2β subunit remains in ∆CK2β-cells, whereas it was unchanged in Mock-cells (Figure S1A,B). We found that several microRNA families were significantly reduced in ∆CK2β-cells (accession number GSE102266, Figure 1A, green bars). The relative miRNA amounts were determined by RT-qPCR using TaqMan probes, including miR-200c, miR-205, miR-141, as well as miR 30b and miR-34b (Figure 1B). The result confirmed the strong decrease of these miRNAs in ∆CK2β-cells. The miR-200 family including hsa-miR-200c, hsa-miR-205 and hsa-miR-141 emerged as the most significantly reduced miRNAs (*p* < 0.001). Direct targets of miR-200c and miR-205 were reported to be the transcription factors ZEB1 and ZEB2 that regulate the epithelial-mesenchymal transition [16,17]. We thus analyzed the protein expression level of ZEB1 in both cell types. Accordingly, we found that ZEB1 expression level was strongly increased in ∆CK2β-cells (Figure 1C, left panel). It has been reported that a miR-30 reduction maintains self-renewal and inhibits apoptosis in breast tumour-initiating cells [18]. Of note, the expression of most members of the miR-30 family including miR-30b, -30c, and -30d, were also reduced in CK2β-depleted cells. A direct target gene of miR-30 is integrin β3 [18]. Consistently, we found the upregulation of the integrin β3 protein in ∆CK2β-cells either by Western blot or by immunofluorescence (Figure 1C). Members of the miR-34 family participate in the regulation of self-renewal and chemotherapeutic resistance of breast cancer cells [19]. When compared to Mock-cells, miR-34 was also significantly reduced in ∆CK2β-cells. Collectively, these data show that ∆CK2β-cells exhibit a decreased expression of specific miRNAs that are all known to regulate de/trans-differentiation, EMT, cell renewal, and invasion.

### 2.2. ∆CK2β-MCF10A Cells Have Increased Expression of Specific miRNAs

We next studied the expression of miR-21, as it is one of the most frequently upregulated miRNAs in solid tumours. In addition, miR-21 is considered to be a typical “onco-miR”, which acts by inhibiting the expression of phosphatases, thus limiting the activity of signaling pathways, such as AKT and MAPK [20]. When compared to Mock-cells, we found that the miR-21 expression was significantly increased in ∆CK2β-cells (Figure 1A,B). As most of the miR-21 targets are tumour suppressors, miR-21 is associated with a wide variety of cancers including breast cancers [21]. Moreover, miR-21 promotes migration and invasion through upregulation of both Sox2 and β-catenin [22], and a loss of polarity associated with an increased expression of collagen type 1 [23]. Interestingly, our transcriptomic analysis showed that different collagen types like collagen I, IV, VI, VII and XIII, were increased more than 3-fold in ∆CK2β-cells as compared to Mock-cells (Table S2). These data were confirmed in the HMEC-hTERT cell line (Figure S3). As mentioned above, integrins are also regulated by miRNAs [24]. Integrin-β3, -α4, and -αV were upregulated whereas integrin-β4 and -β1 were repressed in ∆CK2β-cells (Figure 1D and Table S2). JAG1 is another target of miR-21 that has been shown to be elevated in breast cancer [25]. By RT-qPCR we found that Jagged-1 is repressed in ΔCK2β-MCF10A cells (Figure S1B). Interestingly, miR-1246, mir-21 and miR-210 that have a link with tumour heterogeneity and tumour-initiating cell behaviour, were all induced in ∆CK2β-cells as compared to Mock-cells (Figure 1A,B) [21,26,27,28].

### 2.3. Transcriptomic Analysis

EMT in epithelial cells has been shown to be associated with stem cell traits and chemo-resistance [29]. Comparing ΔCK2β- to Mock-MCF10A cells, we looked further for individual gene expression signatures, using a transcriptomic analysis according to published profiles. Agilent microarrays were performed in duplicates, as previously described [9] (accession number GSE102265) and correlations were done with gene set collections from MSigDB 3.0, as described in Supplementary Figure S1. With this approach, we found that gene signature of Mock-MCF10A cells was correlated with the epithelial profiles while the ΔCK2β-MCF10A signature matched with mesenchymal profiles described in the Charafe, Sarrio, Gotzmann, and Jechlinger collections [30,31,32,33] (Figure S2A). These data extended and confirmed our miRNA analysis together with our previous results [9], and validated the process. Then, using the same approach, the signature of stem cell genes described as upregulated in Boquest, Lim and Pece data [34,35,36], showed a “good” correlation with the one expressed in ΔCK2β-MCF10A cells (Figure S2B). Moreover, this ΔCK2β-upregulated gene signature also correlates with an invasive signature, depicted by Schuetz, Wang, or Poola profiles [37,38,39] (Figure S2C). Altogether, these global analyses prove that ΔCK2β-cells share many genes with stem cells, and suggest that they might exhibit cancer stem cell (CSC)-like properties.

### 2.4. ∆CK2β-MCF10A Cells Have Hallmarks of Stem Cells

#### 2.4.1. CD44/CD24 Stem Cell Markers

**Figure 2:**
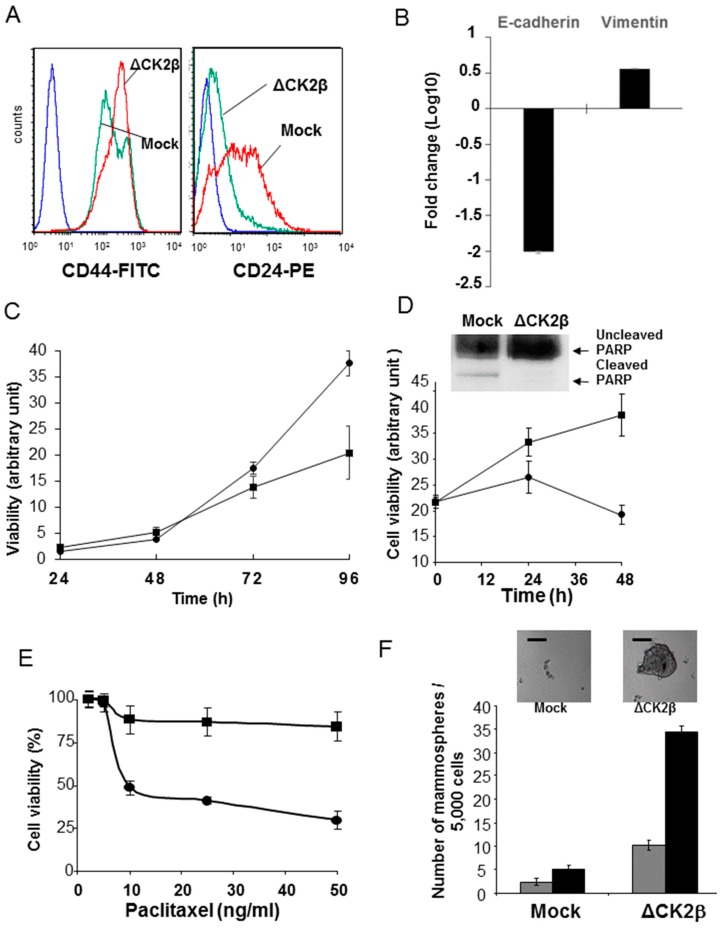
**ΔCK2β-MCF10A cells have properties of cancer stem cells(CSCs) and are drug resistant.** (**A**) FACS analysis of CD24 and CD44 markers in in Mock- and ΔCK2β-cells (Blue line, unlabelled cells; green line, Mock-cells; red line, ΔCK2β-cells). Results are representative of three independent experiments; (**B**) E-cadherin and Vimentin expression levels measured by RT-qPCR. The fold changes compare Mock- to ΔCK2β-cells. *p* < 0.05; (**C**) Cell proliferation kinetic of ΔCK2β- (■) and Mock-MCF10A (●); (**D**) Anoikis: ΔCK2β- (■) and Mock-MCF10A (●) were grown on Poly-HEMA for 48 h. Cell viability was measured with the cell viability-GLO^®^ assay, and apoptosis was visualized by Western blot using anti-PARP antibody; (**E**) Dose-response curves of ΔCK2β- (■) and Mock-MCF10A cells (●) treated with Paclitaxel. Bars denote the standard error (*n* = 5); (**F**) Representative images (top) and quantification (bottom) of mammosphere formation from Mock- and ΔCK2β-cells after first (grey bar) and second (black bar) dissociation steps (scale bar 50 μm).

As it was reported that EMT contributes to the acquisition of cancer stem cells (CSC) traits and drug resistance [29], we further studied the cancer stem cell properties of ΔCK2β-MCF10A cells at the cellular level. Expression of a CD44^high^/CD24^low^ configuration was associated with both human breast CSCs and normal mammary epithelial stem cells [40,41]. Therefore, we examined the consequence of CK2β silencing on CD44 and CD24 expression in ΔCK2β-MCF10A cells using flow cytometry analysis. We found an increase in the CD44^high^/CD24^low^ population in CK2β-depleted cells (95% ± 0.1%), compared to Mock-MCF10A cells (57% ± 1.3%) (Figure 2A). CD44 can contribute to the activation of stem cell regulatory genes and can be a target of these genes [42]. Two connections between CD44 and genes that regulate stem cell characteristics have been described. First, CD44 is a target of the Wnt pathway [43]. Second, stem cells are frequently linked to secondary events such as interaction with a niche, EMT, migration, and apoptosis resistance, which have all been associated with CD44 [44,45]. Our result suggests that a decrease in CK2β expression confers stem cell-like characteristics on epithelial cells. In accordance with the subpopulation of mesenchymal breast cancer stem cells characterized by Liu et al. [46], we found that compared to Mock-cells, ΔCK2β-cells are ALDH and SSEA-1 negative (not shown), and expressed high and low mRNA levels of Vimentin and E-cadherin, respectively (Figure 2B). 

#### 2.4.2. Cell Proliferation and Viability

As already observed for stem cells grown in adherent conditions, cell proliferation was diminished in CK2β-depleted cells (Figure 2C). However, when ΔCK2β- and Mock-MCF10A cells were cultured on Poly-HEMA in the absence of anchorage, ΔCK2β-MCF10A cells showed evidence for increased survival without sign of cell detachment-induced apoptosis (anoikis). In contrast, a decrease of cell viability was observed in control cells, and was found associated with a PARP cleavage (Figure 2D). It has been reported that normal and cancer cell populations became resistant to chemotherapy drug treatment when experimentally induced into EMT [47]. Thus, we tested the viability of Mock- and ΔCK2β-cells treated with a commonly used chemotherapeutic drug, such as Paclitaxel [48]. Figure 2E shows that ΔCK2β-MCF10A cells were more resistant than Mock-MCF10A cells to Paclitaxel.

We further gauge the stemness of cells that have undergone EMT after CK2β depletion, using a mammosphere forming assay in serum free-medium supplemented with EGF and bFGF. We found that both control and CK2β-depleted cells developed spheres or aggregates within seven days, but after their disaggregation, most control cells died, whereas ΔCK2β-cells were still competent to form new mammospheres (Figure 2F).

### 2.5. Cell Positioning and Polarity

**Figure 3:**
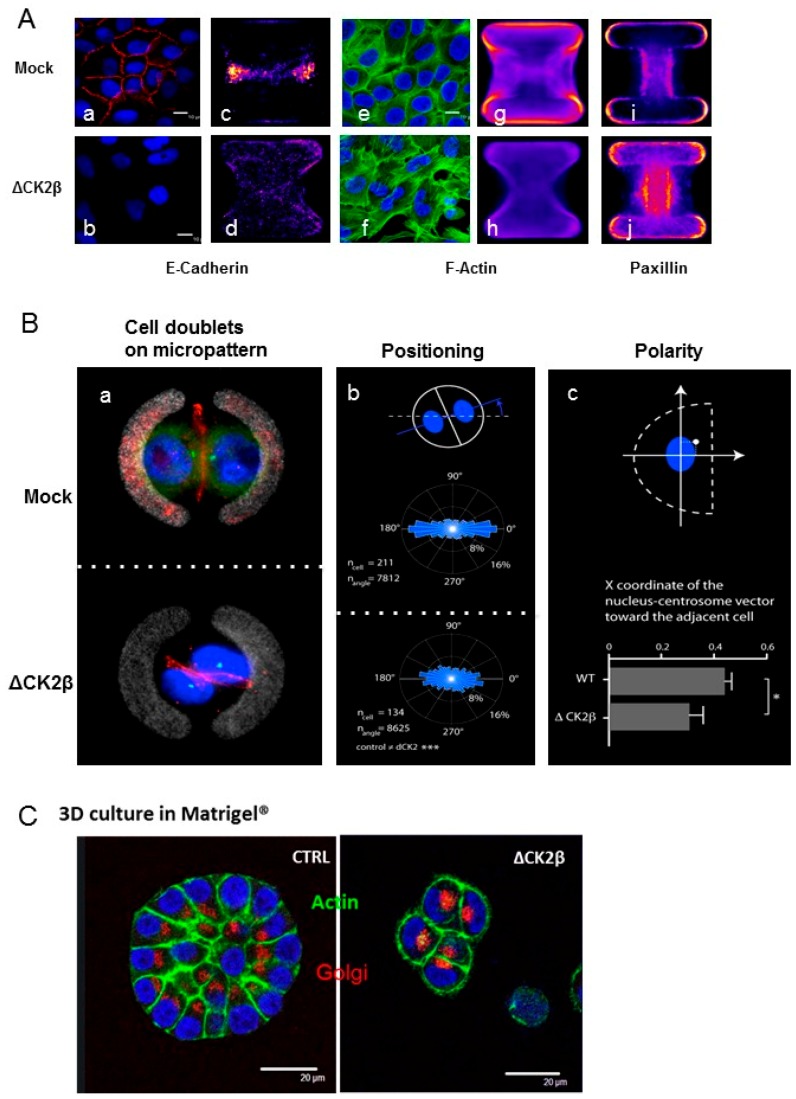
**ΔCK2β- and Mock-MCF10A cell positioning and polarity.** (**A**) Mock and ΔCK2β- MCF10A cells cultured as monolayer (a,b,e,f) or as doublets on H-shaped micropatterns (c,d,g–j) were stained for DNA (blue, a,b,e,f), E-cadherin (red, a–d), F-actin (green, e–h) or paxillin (far red, i,j). Average staining over 20 images on pattern is shown (c,d,g–j). Scale bar, 10 μm; (**B**) (a) Representative image of doublet cells stained for α-catenin (red), centrosome (green), and DNA (blue) on curved H-shaped micropattern; (b) Time-lapse acquisition of control- and ΔCK2β-MCF10A cell doublets on micropattern was performed. Automated movie analysis of Hoechst-stained cells provided the angular distribution of the nucleus–nucleus axis orientation that is represented by graph; (c) The X coordinate of the normalized nucleus-centrosome vector toward the cell-cell junction was calculated. Horizontal bar graph shows quantification of polarity index as previously described [47]; (**C**) Confocal images of 3D culture in Matrigel^®^ for nine days, of Mock- and ΔCK2β-MCF10A cells stained for DNA in blue, F-actin in green, and Golgi apparatus in red. Scale bar, 20 μm.

We previously investigated the cell positioning and polarity of MCF10A cells during TGFβ- induced EMT on H-shaped-micropatterned surfaces that are completely modified [49]. To gain further insight into the polarity changes that arise in ΔCK2β-cells, we first compared the localization of E-cadherin, actin, and paxillin in two-daughter-cell doublets by immunofluorescence (Figure 3A). 

In Mock-MCF10A cells, the cell-cell contacts visualized by E-cadherin Figure 3A(a,c) and cortical F-actin Figure 3A(e,g) were tight. Paxillin, that links integrin to actin filaments, was restricted to the edges of the H-shaped pattern Figure 3A(i). As expected, the E-cadherin staining was lost in ΔCK2β-cells Figure 3A(b,d). Moreover, F-actin was organized as short stress filaments Figure 3A(f) that could be explained by the spatial localization of paxillin all along the adherent pattern Figure 3A(j). To study the position of the two daughter cells on stabilizing curved H-shaped patterns, the spatial coordinates of their nuclei were recorded by time-lapse microscopy during a complete cell cycle and automatically quantified [49]. From the acquired pictures, as exemplified in Figure 3(Ba), the angular distribution of the nucleus–nucleus axis was plotted (Figure 3B(b)), showing that ΔCK2β-cells were in a less stabilized state than Mock-cells. A polarity index has been evaluated, based on nucleus–nucleus axis and normalized nucleus-centrosome vector orientation [50]. Figure 3B(c) showed that this polarity index was significantly decreased in ΔCK2β-cells. These observations are in agreement with our previous data that showed a loss of polarity in CK2β-depleted cells [51]. In contrast, upon EMT induction, TGFβ-treated MCF10A cells behave differently as they underwent a polarity reversal due to both centrosomes and nuclei repositioning [50]. We next compared the behaviour of the two cell lines in 3D Matrigel culture. In these conditions, Mock-MCF10A cells generated fully polarized acinar structures whereas ΔCK2β-MCF10A cells were neither polarized, as visualized by the Golgi position, nor capable to organize in acini (Figure 3C). 

#### Analysis of Cell Phenotypes by Orthotopic Engraftment

**Figure 4:**
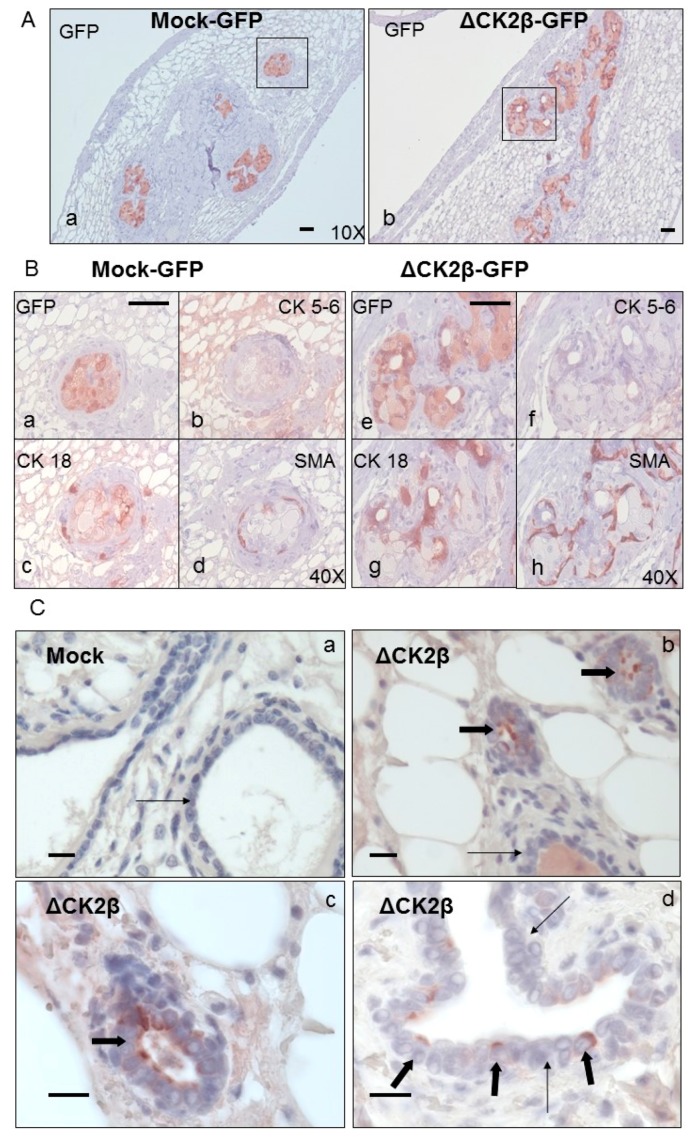
**IHC analysis of ΔCK2β- and Mock-MCF10A cells injected in inguinal mammary fate pad.** (**A**) Three months after injection of GFP-transfected Mock- or ΔCK2β-MCF10A cells in mammary fat pad, the glands were harvested, fixed, paraffin included, and sections were stained with anti-GFP (a and b, respectively); (**B**) High magnification views (40×) of the boxed regions show the staining of GFP (a,e), cytokeratin 5-6 (b,f), cytokeratin 18 (c,g) and αSMA (d,h). Sections were counterstained with hematoxylin; (**C**) Six weeks post-injection of Mock-cells or ΔCK2β-cells, mammary gland sections were immunostained with human specific Cytokeratin 8/18. Sections were counterstained with Hematoxylin. Pictures are representative of different mammary gland sections injected with Mock-cells (a) or ΔCK2β-cells (b–d). Thin arrows indicate mouse mammary epithelial cells and thick arrows human stained luminal cells. Scale bars, 50 μm.

To analyze the cell phenotype in vivo, we injected FACS-sorted GFP-transfected wild type (WT)- or ΔCK2β-MCF10A cells into the inguinal mammary fat pads of nude mice. No tumour formation could be observed three months post-injection. However, IHC analysis of the fat pads showed that for both injected cell types, large islands of viable cells were still visible. The number of GFP-positive areas was increased between two and three months after cell injection (not shown). Compared to Mock-MCF10A cells, fat pads injected with ΔCK2β-cells showed an increased presence of gland-like structures, suggesting that these cells had an organoid-forming activity in this microenvironment (Figure 4A). Interestingly, almost no cytokeratin 5-6 (CK5-6) staining associated with basal cell differentiation, could be detected in those structures, whereas they displayed CK18 and smooth-muscle actin (SMA) staining, which are luminal and myo-epithelial characteristics, respectively. No such structures were observed in mice injected with an equal number of Mock-MCF10A cells (Figure 4B). The same protocol, performed with non GFP-labelled cells, showed that fat pads injected with ΔCK2β-MCF10A cells also displayed gland-like structures in which human mammary epithelial cells were present as visualized by human CK8/18 staining (Figure 4C). 

## 3. Material & Methods

### 3.1. Cell Culture and Retroviral Infection

MCF-10A cells from ATCC-LGS (Molsheim, France) (CRL-10317) are mammary epithelial cells derived from fibrocystic breast tissue from women with no family history of breast cancer and no evidence of disease. HMEC-hTERT were described in [8]. They were both cultured as described [52]. Stable silencing was accomplished by transduction with lentiviruses pLKO1 (Sigma-Aldrich, St. Louis, MO, USA) as described [9]. Mock-cells are MCF10A or HMEC cells transduced with an empty pLKO1 vector.

### 3.2. microRNA Profiling

Total RNA was isolated using Trizol Reagent (Invitrogen, Carlsbad, CA, USA) and was submitted to ProfileXpert core facility (Lyon, France) for microRNA profiling. The samples were hybridized on human v.3 miRNA Agilent array according to manufacturer instructions (Agilent protocol version 2.2), and microarray data analysis was carried out using Feature Extraction software version 10.7 (Agilent, Santa Clara, CA, USA).

For better cross-array comparison, raw data were normalized with the Genespring software version 7.3.1 (Agilent), using two LabelingSpikes-InSignal (DMR_285 and DMR_31a) as internal standard. The threshold of detection was calculated using the normalized signal intensity of negative controls ±3 standard deviation. Spots with signal intensities below this threshold are referred to as (absent) with an arbitrary value of 0.01*, and denoted ‘A’ in the Tables S1 and S2. Quality of processing was evaluated by generating a scatter plot of 11 positive controls. Statistical comparison and filtering were performed using Genespring software 7.3.1 (Agilent). The average signal is averaged between two replicates, and log2 fold change is calculated between ΔCK2β and Mock conditions. The mean and standard deviation of the mean are then calculated to aggregate different probes signal of the same miRNA. 

Both mRNA and miRNA datasets are available under the accession number GSE102267.

### 3.3. Fluorescence-Activated Cell Sorting

FITC-conjugated anti-CD44 antibody (clone G44-26) and PE-conjugated anti-CD24 antibody (clone ML5) were obtained from BD Biosciences (Grenoble, France), and used for FACS analysis in accordance with manufacturer’s protocols. GFP-cells were sorted using a FACS-Calibur (BD Biosciences (Grenoble, France).

### 3.4. Western Blot Analysis

Primary antibodies were anti-actin (Abcam, Cambridge, UK, ab8226), anti-Zeb (Santa Cruz Biotechnology, Heidelberg, Germany, sc-25388), anti-integrin β1, anti- integrin β4 (BD Biosciences, 610467, 611232, respectively), anti- Integrin β3 (Abcam, Ab7167), and anti-PARP (Thermo Scientific, Courtaboeuf, France, 9542). Secondary antibodies were peroxidase-conjugated affinity pure Goat anti-rabbit IgG (#111035003) and peroxidase-conjugated affinity pure goat anti-mouse IgG (#115035003) from Jackson Immuno Research. Cells were lysed in RIPA buffer (10 mM Tris-HCl pH 7.4, 150 mM NaCl, 1% Triton X-100, 0.1% SDS, 0.5% DOC and 1 mM EDTA) containing both protease- and phosphatase-inhibitor cocktails (Sigma-Aldrich; P8340, P2850, P5726). Cell homogenates were quantified using BCA protein assay kit (Thermo Scientific). SDS-PAGE was performed using pre-cast 4–12% gradient gel (Bio-Rad, Hercules, CA, USA). Separated proteins at 20 μg/lane were transferred to PVDF membranes (100 V for 60 min). Blotted membranes were blocked during 1 h at room temperature with saturation buffer (1% BSA in Tris Buffer Saline 10 mM, Tween 0.1% (TBST)), and then incubated with primary antibody diluted in saturation buffer, for 2 h or overnight. After 3 washes with TBST, secondary antibodies were added for 1 h. Luminata Forte Western HRP substrate (Millipore, Billerica, MA, USA) was added and membranes were read with Fusion Fx7 (PerkinElmer, Waltham, MA, USA). Quantification was performed using ImageJ software.

### 3.5. Quantitative Real-Time PCR

One microgram of total RNA prepared for the microarray hybridization was used to generate cDNAs by reverse transcription using the iScript system (Bio-Rad) as recommended by the manufacturer. Real-time PCR was performed using Bio-rad CFX96 apparatus and qPCR Master Mix (Promega, Madison, MI, USA). The values for the specific genes were normalized to the 36B4 and U6. Specific primers sequences are provided in Supplementary Table S3. 

MiRNA levels were measured by RT-qPCR using TaqMan miRNA assays (Applied Biosystems, Foster City, CA, USA). Ten nanograms of tumour total RNA were reverse transcribed using the TaqMan miRNA Reverse Transcription kit and miRNA-specific stem-loop primers (Applied Biosystems). Real-time PCR was performed on the 5’-extended cDNA with Applied Biosystems TaqMan 2× Universal PCR Master Mix, and the appropriate 5 × TaqMan MicroRNA Assay Mix for each miRNA of interest (assay IDs: hsa-miR-205, 4373093; hsa-miR-141, 4373137; hsa-miR-200c, 4395411; hsa-miR-30b, 4373290; hsa-miR-34b, 4395213; hsa-miR-720, 4409111; hsa-miR-21, 000397; hsa-miR-210, 000512; hsa-miR-1246, CTRWENE). Real-time PCR was carried out on C1000 Thermal cycler (CFX96 Real Time system, Bio-Rad) at 95 °C for 10 min, followed by 40 cycles of 95 °C for 15 s and 60 °C for 1 min. Data were analyzed with CFX Manager Software version V1.5.534.0511 (Bio-Rad). The hsa-miR-720 was used as endogenous control for normalization. Normalized expression was calculated using the comparative CT method and fold changes were derived from the 2 − ΔΔCt values for each miRNA.

### 3.6. Characterization of Resistance to Cytotoxic Agents 

All compounds were purchased from Sigma and dissolved in DMSO. MCF10A cells (5000/well) were plated in 100 μL per well in a 96-well plate. One day after seeding, compounds were added in five replicates per concentration. Cell viability was measured after 72 h with Cell viability Glo assay (Promega).

### 3.7. Colony Assay

To measure anchorage-independent growth, cells were detached with trypsin and resuspended in growth medium. Plates were prepared with a coating of 0.75% agarose (Cambrex, East Rutherford, NJ, USA) in growth medium, and then overlaid with a suspension of cells in 0.45% agarose (5 × 10^3^ cells/well). Plates were incubated for 3 weeks at 37 °C and colonies were imaged under microscope.

### 3.8. Mouse Injection

Animal maintenance and experiments were performed in accordance with the animal care guidelines of the European Union and French laws. Six-week old female Athymic nude mice (Charles River laboratories) were injected with 10^6^ modified MCF10A cells into a fat pad of #4 mammary gland. 

### 3.9. Histological and Immunohistochemical Analysis

Mammary glands were fixed in 4% paraformaldehyde and embedded in paraffin. Microtome sections (5 mm thick) were stained with H&E for histological analysis. Expression of the transduced GFP was analyzed by standard immuno-histochemistry (IHC) using the anti-GFP mouse monoclonal antibody (Abcam, Ab 13970), detected with a biotin-conjugated anti-mouse IgG antibody and an avidin-biotin-peroxidase complex (Vector Laboratories). The differentiation status of tissues was determined using CK5-6 (Chemicon, Millipore, MAB 1620, Billerica, MA, USA) CK18 (Epitomics, Burlingame, CA, USA, 1433-1) and SMA (DAKO, Agilent, M0851, Santa Clara, CA, USA) antibodies.

### 3.10. Gene Expression Microarray Analysis 

Single-sample GSEA (ssGSEA), an extension of Gene Set Enrichment Analysis (GSEA) calculates separate enrichment scores for each pairing of a sample and gene set. Each ssGSEA enrichment score represents the degree to which the genes in a particular gene set are coordinately up- or down-regulated within a sample. This analysis has been done with the GenePattern software [53]. (http://software.broadinstitute.org/cancer/software/genepattern/modules/docs/ssGSEAProjection/4).

### 3.11. Cell Micropatterning

Micropatterns were fabricated and cell-cell positioning was analyzed as previously described [49]. The polarity index was measured as described in [50].

### 3.12. Immunofluorescence 

Cells were fixed as previously described [47], and incubated with anti-integrin β1, anti-integrin β4, anti-paxillin, anti-E-cadherin (BD Biosciences, 610467, 611232, 6100052, and 610181, respectively), anti-integrin β3, anti-Giantin (Abcam Ab7167 and 24586 respectively), and anti-α-catenin (B52975; Calbiochem), for 1 h, and then incubated with the corresponding secondary antibodies and FITC-phalloidin (Invitrogen) at 1 μg/mL for 30 min.

## 4. Conclusions

It has been reported that the EMT programs which control normal mammary stem cells and cancer stem cells are likely to differ in the activation of distinct signaling pathways [54]. Together, our data provide evidence that the downregulation of CK2β expression, observed in a subtype of breast tumours, can promote the acquisition of characteristics commonly associated with the CSC phenotype in vitro. However, since ΔCK2β-MCF10A cells were deficient in tumour-initiating ability, we suggest that under-expression of CK2β has a profound impact on both the plasticity and the stemness of breast epithelial cells. In particular, the results from Figure 4 show that micro-environmental cues are sufficient to re-direct cells that exhibit stem cell traits to acquire an organoid-forming activity with an absence of malignant transformation. Since EMT supports the induction stem-cell phenotype, identifying the CK2 substrates whose phosphorylation is modulated in CK2β-depleted cells or CK2α overexpressing cells, will improve the discovery of new specific markers furthering understanding breast cell stemness. 

## Supplementary Materials

The following are available online at http://www.mdpi.com/2072-6694/9/9/114/s1. Figure S1: Expression analysis of CK2β and JAG1, Figure S2: Gene expression microarray analysis, Figure S3: Modulation of miRNA-targets in ΔCK2β-HMEC-hTERT cells, Table S1: miRNA expression profile of Mock and ΔCK2β-MCF10A cells, Table S2: Normalized mRNA expression profile of Mock and ΔCK2β-MCF10A cells, Table S3: Primers used for RT-qPCR.
